# Genome-Wide Association Study Reveals Candidate Genes for Root-Related Traits in Rice

**DOI:** 10.3390/cimb44100301

**Published:** 2022-09-21

**Authors:** Jun Xiang, Chaopu Zhang, Nansheng Wang, Zhaojie Liang, Zheng Zhenzhen, Lunping Liang, Hongyan Yuan, Yingyao Shi

**Affiliations:** College of Agronomy, Anhui Agricultural University, Hefei 230000, China

**Keywords:** epistatic interactions, GWAS, QTLs, root-related traits, rice

## Abstract

Root architecture is a determinant factor of drought resistance in rice and plays essential roles in the absorption of water and nutrients for the survival of rice plants. Dissection of the genetic basis for root structure can help to improve stress-resistance and grain yield in rice breeding. In this study, a total of 391 rice (*Oryz asativa* L.) accessions were used to perform a genome-wide association study (GWAS) on three root-related traits in rice, including main root length (MRL), average root length (ARL), and total root number (TRN). As a result, 13 quantitative trait loci (QTLs) (*qMRL1.1*, *qMRL1.2*, *qMRL3.1*, *qMRL3.2*, *qMRL3.3*, *qMRL4.1*, *qMRL7.1*, *qMRL8.1*, *qARL1.1*, *qARL9.1*, *qTRN9.1*, *qTRN9.2*, and *qTRN11.1*) significantly associated with the three traits were identified, among which three (*qMRL3.2*, *qMRL4.1* and *qMRL8.1)* were overlapped with *OsGNOM1*, *OsARF12* and *qRL8.1*, respectively, and ten were novel QTLs. Moreover, we also detected epistatic interactions affecting root-related traits and identified 19 related genetic interactions. These results lay a foundation for cloning the corresponding genes for rice root structure, as well as provide important genomic resources for breeding high yield rice varieties.

## 1. Introduction

Rice (*Oryz asativa* L.) is one of the most important crops, providing staple food for more than half of world population [1]. With the rapid growth of population and increasing scarcity of water resource, abiotic stresses have posed great threats to crop yield, making it an important task to increase and stabilize food production [2]. The foundation of the continued improvement of rice cultivars is the rich genetic diversity within domesticated populations and wild relatives [3,4]. Rice is known for its rich species diversity, *Xian* (*indica*)-*Geng* (*japonica*) subspecies differentiation, and subgroup differentiation [5,6,7,8,9]. In addition, rice has undergone decades of intensive improvement since the Green Revolution of the 1950s. Ancient human domestication and modern breeding share a common factor of both selection to improve productivity and adaptation to specific environments. Strong root structure is important for the growth, yield, and stress resistance, as well as optimization of nutrient and water absorption of rice [10,11]. Therefore, it is of great significance to understand the genetic mechanism of rice root structure [11].

Root growth is a complex process affected by multiple genes, environmental factors, and hormones [12], such as auxin (IAA) and cytokinin (CTK) [13]. Indole-3-acetic acid is the main active form of IAA in plants. Mutations in genes related to IAA metabolism, transport, or signal transduction will significantly alter the root development of rice. *OsAUX1* is involved in auxin polar transport. Under hydroponic conditions, the *OsAUX1* mutant exhibited longer main roots, shorter root hairs, and lower sensitivity to IAA and synthetic auxin compared with the wild type [14], while opposite phenotypes were observed in *OsAUX1* overexpression lines [15]. *OsYUCCA1* plays an important role in the tryptophan-dependent IAA biosynthesis pathway. Overexpression of *OsYUCCA1* increased the IAA level, resulting in a significant increase in rice coronal roots [16]. Lack of water or nutrients retards plant growth, and optimization of the root structure can mitigate the effect of various stresses. Auxin response factor *OsARF12* is a limiting factor in root structure. Knockout of *OsARF12* decreased the expression of auxin synthesis genes *OsYUCCAs* and auxin efflux vectors *OsPINs* and *OsPGPs*, resulting in a shortened main root length in rice [17]. *OsGH3-2* encodes an enzyme that catalyzes the binding of IAA for amino acids. Overexpression of *GH3-2* reduced the number of coronal roots and resulted in sparse root hairs [18]. Contrary to that of auxin, overexpression of cytokinin biosynthesis gene (*OsIPT*) and cytokinin response gene response regulator 6 (*OsRR6*) resulted in root dysplasia [19,20]. Metallothionein gene *OsMT2b* was downregulated by cytokinin. Compared with those of wild type plants, the growth and root formation of *OsMT2b* RNAi transgenic plants were severely inhibited [21]. The WUSCHEL-related homeobox gene *WOX11* integrates auxin and cytokinin signal transduction, and directly inhibits the expression of type A cytokinin response regulator RR2 in coronal root primordium and promotes coronal root growth [22].

Certain phytohormone-related genes also play an important role in regulating the growth of primary roots in rice. For example, *SLR1* encodes a DELLA protein, which is a negative regulator of gibberellin (GA) signal transduction and can also integrate and amplify defense signals mediated by salicylic acid (SA) and jasmonic acid (JA). The function-loss mutation of *SLR1* reduced the number of rice roots [23]. *OsRMC*, a plasma membrane protein of DUF26 signal peptide, participates in JA signal transduction, mediates rice root development, and negatively regulates root curling [24]. Ethylene can promote auxin synthesis and regulate plant root cell elongation [25,26]. However, it remains unclear whether ethylene regulates root development through auxin accumulation and transport in rice.

In recent years, the development of gene cloning technology, rice genome research, and molecular marker technology has provided great technical and theoretical support for quantitative trait loci (QTL) mapping and breeding research on rice roots. Numerous genes related to rice root traits have been discovered and cloned. For example, the short root phenotype of rice was found to be controlled by an invisible gene *OsCYT-INV1*. The substitution mutation of a nucleotide led to the change of an amino acid in the encoded protein from glycine to arginine, thus controlling the growth of rice short roots [27]. The *OsSPR1* gene encodes a new mitochondrial protein that regulates rice root length [28]. *REL2* [29] and *OsWRKY74* [30] affect the number and length of adventitious roots, and *OsCYP2* [31] controls lateral root growth.

Linkage analysis and association analysis are important methods to analyze quantitative traits and are also widely used in the study of rice roots. Many quantitative trait loci (QTLs) of rice root traits have been identified by the traditional parental mapping method. For example, PHOSPHORUS UPTAKE 1 (*Pup1*) was identified as a QTL related to phosphorus deficiency tolerance in the autumn rice variety Kasalash. Sequencing revealed that this locus has a Pup1-specific protein kinase gene *PSTOL1*, which is not present in modern rice varieties. *PSTOL1* is an early root growth enhancer that enhances the ability of plants to acquire phosphorus and other nutrients [32]. QTL detection demonstrated that the main QTL on chromosome 9 is DEEPER ROOTING 1 (*DRO1*). *DRO1* is negatively regulated by auxin and affects cell elongation at the root tip, resulting in asymmetric root growth and downward bending by gravity [2]. The mapping population consisting of 480 lines from the hybridization between Dular and IR64-21 was also detected. The root growth angle related to *qRT9.1* and seedling root length, root dry weight, and shoot number associated with *qRT5.1* were identified [33]. Recently, due to the emergence of next generation sequencing, genome-wide association study (GWAS) has rapidly developed into a powerful tool for crop genetic variation analysis [34], which has been successfully applied to different traits such as yield, metabolism, and stress response in rice [35,36,37].

In GWAS, the statistical association between species-specific traits and single nucleotide polymorphism (SNP) is used to test the association between traits and loci [38,39]. Since the adjacent regions of related loci can be inherited together, more loci can be associated with the phenotype. Compared with QTL mapping, GWAS can explain more extensive genetic variations [40]. For example, GWAS analysis was performed on 795 rice accessions, and 44 and 97 loci for root length and diameter were identified, respectively [41]. Zhao [42] revealed that glucosyltransferase *OsIAGLU* regulates rice root growth in 178 rice accessions through GWAS. GWAS has also been used to identify the related genes *Nal1*, *OsJAZ1* and *Nced* of three root traits [43,44]. Although several genes related to rice root length have been cloned, more candidate genes are required to meet the needs of rice breeding. Epistasis plays an important role in complex and multi-group genetics [45]. Some methods and software packages have been developed to detect the epistatic interaction in GWAS, including iLOCi [46], BiForce Toolbox [47], BOOST [48], and 3VmrMLM [49] for genome-wide research.

In this study, 391 rice accessions were used to investigate the main root length (MRL), total root number (TNR), and average root length (ARL) of rice. Combined with rice resequencing genotypes, GWAS of rice was performed using the general linear model (GLM) and mixed linear model (MLM). The newly developed 3VmrMLM software package was used to analyze the interaction of GWAS results. SNPs related to rice root length traits were identified, and the candidate genes were analyzed to further investigate the epistatic interaction between GWAS loci. In addition, the abundant genetic heterogeneity and epistatic interaction among root traits genes in rice were revealed. The findings provide an excellent resource for genetic improvement of rice root structure.

## 2. Materials and Methods

### 2.1. Plant Materials

A total of 391 rice accessions were obtained from 3000 Rice Genome Project (3KRGP) in 36 countries and regions and planted in paddy fields of Anhui Agricultural University (31.93 N, 117.39 E) in 2021 (Table S1) [50]. Each line was planted in three rows with eight individuals per row with spacing of 17 × 33 cm. Field management was carried out according to the local standard practices [51]. Seeds were harvested at 30 to 35 days after heading at maturity considering the inconsistent heading date, which were then dried, and seal preserved. The germination test was carried out uniformly after completion of the harvest of all the required materials.

### 2.2. Evaluation of Main Root Length, Total Number of Roots, and Average Root Length

The seeds of all 391 rice accessions were dried in a 42 °C oven for 7 days to break dormancy. Twenty mature and full seeds were selected, washed with distilled water, and germinated in a 32 °C incubator for 64 h. Eight seeds of each variety germinated evenly were placed in the germination box, and the germination test was carried out at 30 °C in the light incubator. On the 10th day of germination, the root length and total root number (TRN) of each material were measured [52]. The length the longest root was taken as the main root length (MRL), and the average root length (ARL) was calculated as the sum of root length/TRN. The phenotypic mean measured by each cultivar was used for subsequent analysis. The R packages of ‘corrplot’ and ‘ggplot2’ were used to draw the correlation coefficient matrix and violin graphs of phenotypic data across subgroups.

### 2.3. Genotyping and Population Structure Analysis

The resequencing data of 391 rice accessions have been published in NCBI, and the variation site information can be obtained by SNP-Seek database (https://snp-seek.irri.org/index.zul, accessed on 15 January 2021) [50,53]. We used PLINK (version 1.9) [54], MAF (0.05), and GENO (0.2) to further filter SNPs and screened 1,821,713 SNPs with minor allele frequency (MAF) > 5% and missing data rate (MDR) < 0.2 (Supplementary Figure S1). The distance matrix was constructed using VCF2Dis (https://www.opensourceagenda.com/projects/vcf2dis, accessed on 20 February 2021) based on filtered files, and the phylogenetic tree was constructed using iTol (https://itol.embl.de/, accessed on 15 April 2021). GCTA [55] (version 1.93) was used for principal component analysis (PCA) to estimate the number of subgroups in the GWAS panel. In order to avoid the influence of linkage SNP in the process of population structure analysis, SNP loci were filtered according to linkage disequilibrium (LD), and unlinked SNP was retained to obtain 45,311 SNPs. The population structure analysis was carried out using Admixture (https://speciationgenomics.github.io/ADMIXTURE/, accessed on 18 May 2021) software, and the genetic structure of the whole population was predicted. In order to construct the kinship matrix required for the mixed linear model as the covariance matrix, this study used the software Tassel (version 5.0) [56] to analyze the kinship. The population structure diagram, PCA diagram, and kinship diagram were drawn by R.

### 2.4. Genome-Wide Association Study

A total of 1821713, 1184751, and 882400 SNPs with MAF > 5% and MDR < 0.2 were screened for association analysis of the whole population, *Xian* rice population, and *Geng* rice population. MRL, TRN, and ARL of the whole population, *Xian*, and *Geng* rice were analyzed by GWAS using R software package rMVP [57]. Two models, GLM (only considering population structure) and MLM (considering population structure and relative kinship), were used to analyze the association between phenotype and genotype datasets. The suggested *p* value of dominance was 1.0 × 10^−^^5^ to control the genetic false positive error rate of the population. The Manhattan and quantile graphs of GWAS results were created using ‘rMVP’ package of R. The LD attenuation of different populations, different chromosomes, and different genome regions in the same chromosome was different. Hence, for a certain site, the resolution is determined by the local LD attenuation level. The average LD decay distance of accession resources was analyzed by software PopLDdecay (version 3.4), and the screening standard was the physical distance when R^2^ was reduced to half of the maximum. The LD decay distance of the whole population, *Xian* population, and *Geng* population was 350 kb, 125 kb and 346 kb, respectively (Supplementary Figure S2), which is consistent with previous findings in rice LD [34]. Therefore, the associated signal may appear in the upstream 350 kb or downstream 350 kb of the pathogenic gene. Hence, we chose a 700 kb distance as the recognition overlap marker-trait associated signal range.

### 2.5. Selection and Analysis of Candidate Genes

In order to identify candidate genes related to MRL, TRL, and ARL, the rice genome annotation project (http://rice.plantbiology.msu.edu, accessed on 15 January 2021) was used to search candidate genes in the 700 kb genomic region of selected important SNPs. Among all candidate genes, four types of genes, including expression protein, hypothetical protein, retrotransposon, and transposon, were excluded. ANNOVAR software was used to further select the polymorphism significantly related to GWAS trait variation and predicted to induce amino acid exchange or change splicing connection as candidate genes [58].

### 2.6. Haplotype Analysis

SNP data of candidate genes were extracted based on genotype of SNP with MAF > 0.05. This dataset only contained double-allele SNPs. Further haplotype analysis excluded heterozygotes and missing alleles. Haplogroups consisting of fewer than 10 accessions were deleted. For the genes found in QTLs, haplotype analysis only used the non-synonymous SNPs in the coding region of these genes for haplotype analysis of R, and the student t test was used to determine whether this locus could cause changes in rice root traits. R was used to visualize the results.

### 2.7. Epistatic Interaction Analysis

We selected 10,801, 4227, and 6056 SNPs from GWAS (−Log_10_ (*p*) > 3.0) of MRL, TRN, and ARL under GLM for epistasis interaction analysis. Epistasis method of 3 V multi-locus random SNP effect mixed linear model (3VmrMLM) [49] was used to detect epistatic interaction of main effect QTLs.

## 3. Results

### 3.1. Population Structure and Root Traits

**Figure 1:**
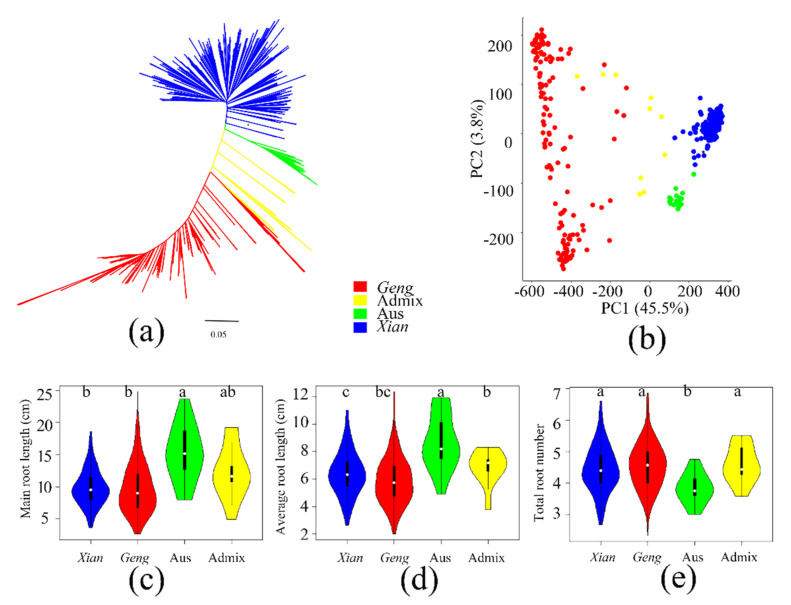
Phenotypic diversity of population structure and root traits of 391 rice accessions. (**a**) Based on the neighbor-joining tree of 391 samples, the filtered SNPs were calculated by a simple matching distance matrix; (**b**) Principal component analysis based on SNPs clearly distinguished *Geng*, *Xian*, Aus, and admix accessions, indicating different genetic backgrounds; Distribution of (**c**) main root length, (**d**) average root length, and (**e**) total root number among different rice populations. Different letters on the violins indicate statistically significant differences at *p* < 0.05 based on Tukey’s test.

A phylogenetic tree and principal component analysis were used to analyze the genetic structure of SNPs in 391 rice accessions. The phylogenetic tree showed that 391 accessions were clustered into four groups (Figure 1a). Similar results were obtained by principal component analysis, and the first two principal components can explain most of the genetic variations between accessions (Figure 1b). For the first two components, most accessions were clustered into four groups. Then, the 391 rice accessions were divided into four groups, namely *Xian* (*n* = 213), *Geng* (*n* = 147), Aus (*n* = 21), and admix population (*n* = 10) (Table 1 and Supplementary Table S1).

As shown in Table 1, the mean MRL of 391 rice accessions was 10.11 cm, among which the mean MRL of Aus and *Xian* rice was 15.14 cm and 9.71 cm, respectively. There was no significant difference in mean MRL between *Xian* rice and *Geng* rice (Figure 1c and Table S2). For ARL, the average value of *Xian* rice was 5.88 cm, whereas that of Aus was 8.68 cm, and that of *Geng* rice and admix was 6.39 cm and 6.88 cm, respectively (Figure 1d). The average TRN of Aus was 3.83, which was significantly different from that of other three clusters (Figure 1e). The variation of MRL, ARL, and TRN in rice was extensive with a normal distribution (Supplementary Figure S3), indicating that the root length traits are controlled by multiple genes, which provides a basis for further understanding the genetic structure of roots.

### 3.2. Genome-Wide Association Study of MRL, ARL, and TRN Traits

**Figure 2:**
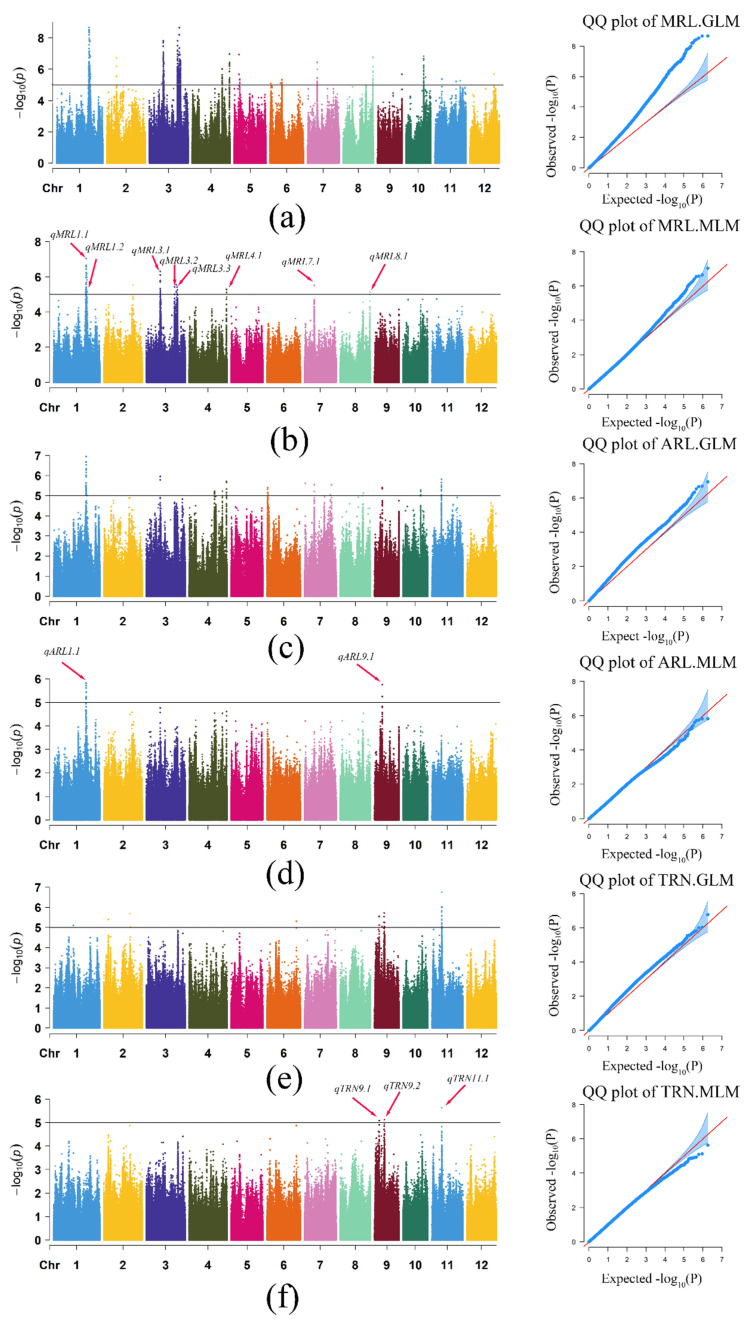
Genome-wide association plots of MRL, ARL, and TRN in rice population plotted using the general linear model and mixed model methods. The Manhattan map of genome-wide scans shows the −log_10_ (*p*) values corresponding to the location of each of 12 chromosomes. Black solid lines represent the whole genome significant threshold *p* = 5.0 × 10^−5^. Red arrows indicate QTLs (*qMRL1.1*(Chr1_30384232) *qMRL1.2*(Chr1_ 31258476) *qMRL3.1*(Chr3_13180766) *qMRL3.2*(Chr3_26623430) *qMRL3.3*(Chr3_28214674) *qMRL4.1*(Chr4_34983993) *qMRL7.1*(Chr7_9145116) *qMRL8.1*(Chr8_27836609) *qARL1.1*(Chr1_30377471) *qARL9.1*(Chr9_7252407) *qTRN9.1*(Chr9_4342978) *qTRN9.2*(Chr9_9220591) and *qTRN11.1*(Chr11_9292368)) co-localized by GLM and MLM. The horizontal axis in the quantile-quantile (QQ) plot represents the expected value of the −log_10_ transformation, whereas the vertical axis represents the observed value of the −log_10_ transformation. Manhattan plot and QQ plot of MRL (**a**) in GLM (**b**) and MLM; Manhattan plot and QQ plot of ARL in GLM (**c**) and MLM (**d**); Manhattan plot and QQ plot of TRN in GLM (**e**) and MLM (**f**).

The general linear model (GLM) and mixed linear model (MLM) were used to conduct GWAS on three root traits (MRL, ARL, and TRN). Considering the attenuation distance of linkage disequilibrium (LD) in rice, adjacent SNPs with spans less than 700 kb were defined as single QTLs, and the SNP with the lowest *p* value was used as the lead SNP to reduce the redundancy of association signals of different traits. The results showed that 22, 11, and 12 QTLs detected by GLM were significantly correlated with MRL, ARL, and TRN, respectively. A total of 9, 2, and 3 QTLs were detected for MRL, ARL, and TRN using the MLM approach (−log_10_ (*p*) ≥ 5), respectively (Supplementary Tables S3 and S4). The GLM of MRL contains 88.9% of QTLs of MLM, and all QTLs of MLM of ARL and TRN are located in GLM. GLM had a high false positive rate, although MLM could effectively reduce the false positive rate. To effectively reduce the false positives, we selected the QTLs co-located by MLM and GLM as candidate regions (Table 2). In general, eight QTL regions were determined to be significantly correlated with MRL, including *qMRL1.1* and *qMRL1.2* on chromosome 1, *qMRL3.1*, *qMRL3.2*, and *qMRL3.3* on chromosomes 3, *qMRL4.1* on chromosome 4, *qMRL7.1* on chromosome 7, and *qMRL8.1* on chromosome 8 (Figure 2a,b). QTLs associated with ARL were *qARL1.1* on chromosome 1 and *qARL9.1* on chromosome 9 (Figure 2c,d). Three loci, including *qTRN9.1* and *qTRN9.2* on chromosome 9 and *qRN11.1* on chromosome 11, were significantly associated with TRN (Figure 2e,f). One QTLs (*qMRL10.1*) were identified for the *Geng* panel and ten QTLs (*qMRL1.1*, *qMRL1.2*, *qMRL3.1*, *qMRL3.2*, *qMRL3.3*, *qARL2.1*, *qARL4.1*, *qARL12.1*, *qTRN9.1* and *qTRN11.1*) were identified for the *Xian* panel (Supplementary Table S5). Five MRL-related QTLs (*qMRL1.1*, *qMRL1.2*, *qMRL3.1*, *qMRL3.2* and *qMRL3.3*) and one TRN-related QTL (*TRN11.1*) were detected in the *Xian* rice population, and no QTL co-located with ARL was detected (Supplementary Figure S4 and Table S5). In *Geng* rice population, no QTL was detected to be co-located with that of the whole population (Supplementary Figure S5 and Table S5). There was no significant difference between *Xian* and *Geng* in three phenotypic traits (Figure 1c–e). In our study, the number of *Xian* rice was greater than that of japonica rice. Through GWAS of *Xian* and *Geng* rice subgroups, it can be concluded that *Xian* rice provides the main genetic variation for rice root length.

### 3.3. Identification of Candidate Genes for MRL

**Figure 3:**
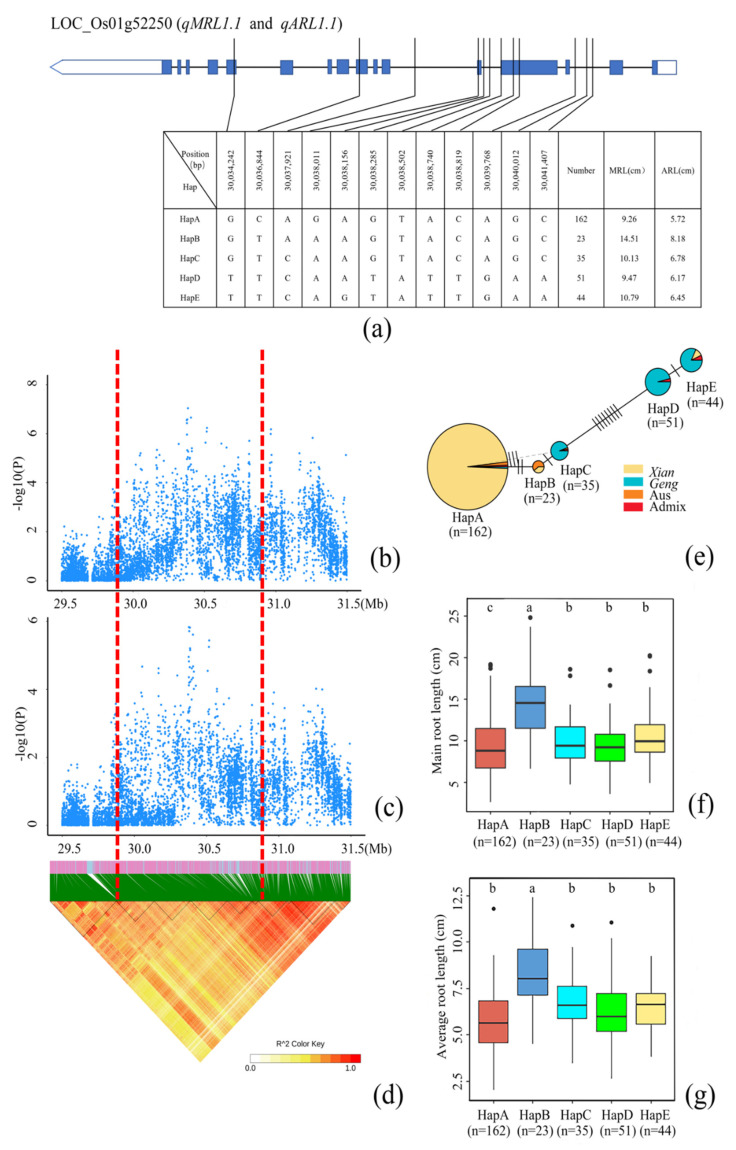
Identification of candidate genes for MRL. (**a**) Based on 12 SNPs observed in all evaluated rice accessions, five haplotypes of LOC_Os01g52250 were identified. In the gene structure diagram of LOC_Os01g52250, the promoter is indicted by white frame; the exon is represented by blue frame; and the intron and intergenic region are marked by black lines. A thin black line represents the genomic location of each SNP. Haplotypes with fewer than 10 accessions are not shown. MRL (**b**) and ARL (**c**) based on single polymorphism and LD thermal map of local Manhattan map (**d**), around the peak on chromosome 1. Red dotted lines represent candidate regions for associated SNPs; (**e**) Haplotype network diagram of LOC_Os01g52250. In each haplotype network, two adjacent haplotypes are separated by mutation changes. Based on MRL (**f**) and ARL (**g**) of LOC_Os01g52250 haplotype, differences between the haplotypes were statistically analyzed using Tukey’s test.

There were eight QTLs (*qMRL1.1 qMRL1.2 qMRL3.1 qMRL3.2* [59] *qMRL3.3 qMRL4.1* [60] *qMRL7.1*, and *qMRL8.1* [42]) associated with MRL, among which five were novel QTLs (*qMRL1.1*, *qMRL1.2*, *qMRL3.1*, *qMRL3.3*, and *qMRL7.1*). Further annotation with the ANNOVAR software screened the genes that can predict the induction of amino acid exchange or change the splicing point. In *qMRL1.1*, *qMRL1.2*, *qMRL3.1*, *qMRL3.3*, and *qMRL7.1*, there were respectively two, three, one, fifteen, and ten genes causing non-synonymous mutations (Supplementary Table S6). Interestingly, the lead SNPs of *qMRL1.1* and *qARL1.1* were 7 kb apart, sharing the same candidate genes (Supplementary Figure S6). The QTL region contained two candidate genes annotated as starch synthase (LOC_Os01g52250) and pathogen-related protein (LOC_Os01g53090). We then focused on LOC_Os01g52250 and performed a haplotype analysis. Twelve non-synonymous SNP loci were found in the region (30.034–30.042 Mb), and haplotypes with fewer than 10 accessions were deleted, which formed five haplotypes (Figure 3a–c). Multiple comparisons revealed that HapB had significantly longer MRL and ARL than other four haplotypes (Figure 3g,f). HapA was the main haplotype of *Xian* rice, with nearly exclusive distribution in *Xian* rice, which might be related to the significant reduction of MRL and ARL. The major difference between HapA and HapB was in two non-synonymous mutations. HapB was mainly distributed in Au’s rice and *Xian* rice, which was related to the significant increase in MRL and ARL in Aus rice. HapC was mainly distributed in *Geng* rice, and the major difference from HapB was a non-synonymous mutation (Figure 3e). These results explained the response of different subpopulations to MRL and ARL variations. Similarly, we also focused on the gene LOC_Os03g22830 in the *qMRL3.1* region that can cause amino acid changes, which was annotated as a zinc finger protein containing the C3HC4 domain. LOC_Os03g22830 had three haplotypes. The accessions carrying HapC showed longer MRL than those carrying HapA or HapB (Supplementary Figure S7). In the *qMRL3.3* region, it is worth noting that the reported lipoxygenase gene OsLOX5 was proved to be the first key enzyme in JA biosynthesis pathway. OsLOX5 had five major haplotypes, with HapA, HapD, and HapE mainly existing in Aus, *Geng* and *Xian* rice, respectively (Supplementary Figure S8). Multiple comparisons revealed that HapA had significantly higher MRL than HapD and HapE, indicating that OsLOX5 is a causal gene in MRL. Similarly, OS-ETR4 was a cloned gene in the *qMRL7.1* region, which mainly had four haplotypes. The accessions carrying HapC and HapD had significantly longer MRL than those carrying HapA and HapB (Supplementary Figure S9). These results indicated that OS-ETR4 is also involved in the regulation of MRL in rice.

### 3.4. Identification of Candidate Genes for ARL

**Figure 4:**
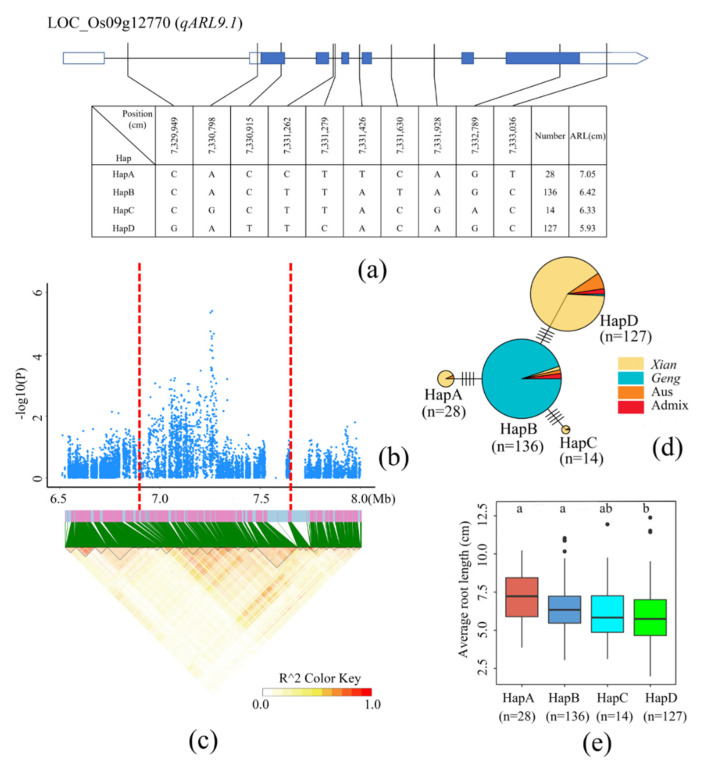
Identification of candidate genes for ARL; (**a**) Based on 10 SNPs observed in all evaluated rice accessions, four haplotypes of OsMYBc (LOC_Os09g12770) were identified. In the gene structure diagram of OsMYBc, the promoter is indicated by white frame; the exon is represented by blue frame; and the intron and intergenic regions are marked by black lines. A thin black line represents the genomic location of each SNP. Haplotypes with fewer than 10 accessions are not shown. ARL (**b**) based on single polymorphism and LD thermal map of local Manhattan map (**c**), around the peak on chromosome 9. Red dotted lines represent candidate regions for associated SNPs. (**d**) Haplotype network diagram of OsMYBc. In each haplotype network, two adjacent haplotypes are separated by mutation changes. Based on ARL (**e**) of OsMYBc haplotype, differences between the haplotypes were statistically analyzed using Tukey’s test.

In the two loci (qARL1.1 and qARL9.1) significantly associated with ARL, the candidate region in chromosome 9 was mapped from 6862 to 7642 kb (Figure 4a–c). This region contained eight candidate genes for nonsynonymous mutations in the exon region (three reported genes, two annotated as plant proteins with unknown functional domains, and three annotated as enzymes) (Supplementary Table S6). We then mainly focused on the known gene OsMYBc, which can bind to the AAANATNC sequence of OsHKT4 promoter to regulate its expression, thereby affecting the salt tolerance of rice. There were four haplotypes (Figure 4a). The accessions carrying HapA had a significantly longer ARL than the accessions carrying HapD (Figure 4e). The difference between HapD and HapB was in four non-synonymous mutations. HapD was mainly distributed in *Xian* rice, and HapB was mainly distributed in *Geng* rice (Figure 4d). The ARL of *Xian* rice carrying HapD was significantly lower than that of *Geng* rice carrying HapB (Figure 4e). Based on these data, it could be speculated that OsMYBc is a functional gene at this locus and is involved in the regulation of rice ARL.

### 3.5. Identification of Candidate Genes for TRN

**Figure 5:**
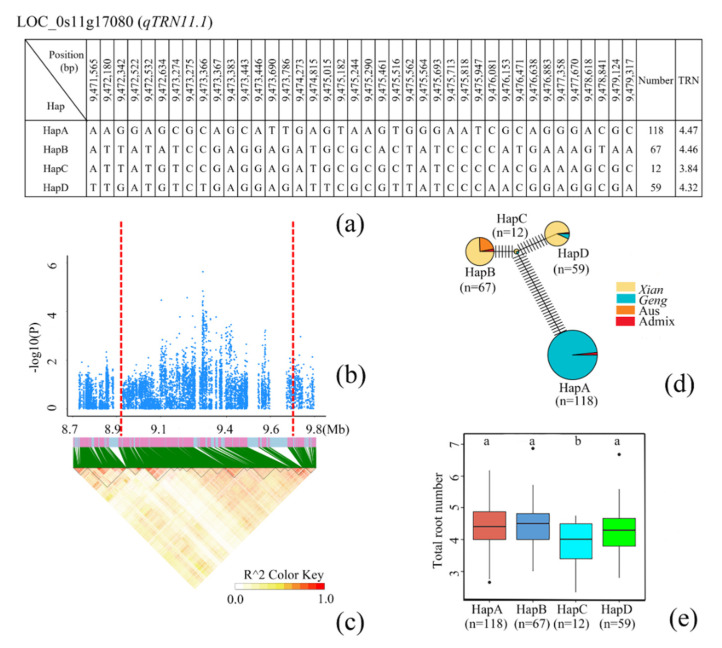
Identification of candidate genes for TRN. (**a**) Based on 40 SNPs observed in all evaluated rice accessions, four haplotypes of OsMPK15 (LOC_Os11g17080) were identified. In the gene structure diagram of OsMPK15, the promoter is indicated by white frame; the exon is represented by blue frame; and the intron and intergenic regions are marked by black lines. A thin black line represents the genomic location of each SNP. Haplotypes with fewer than 10 accessions are not shown. TRN (**b**) based on single polymorphism and LD thermal map of local Manhattan map (**c**), around the peak on chromosome 9. Red dotted lines represent candidate regions for associated SNPs. (**d**) Haplotype network diagram of OsMPK15. In each haplotype network, two adjacent haplotypes are separated by mutation changes. Based on TRN (**e**) of OsMPK15 haplotype, differences between the haplotypes were statistically analyzed using Tukey’s test.

GWAS identified three TRN-related QTLs (qTRN9.1, qTRN9.2 and qTRN11.1), and screened eight (qTRN9.1), one (qTRN9.2), and 17 (qTRN11.1) non-synonymous mutation genes in 350 Kb upstream and downstream of the lead SNP in the three QTLs, respectively (Supplementary Table S6). According to the known genes controlling rice root traits, we focused on LOC_Os09g07900 in the qRN9.1 region, LOC_Os09g15400 in the qTRN9.2 region, and LOC_Os11g17080 in the qTRN11.1 region. A haplotype analysis of the known gene OsMPK15 (LOC_Os11g17080) showed that OsMPK15 was located at 179 kb of the lead SNP of qTRN11.1 (Figure 5b,c). OsMPK15 had four major haplotypes, and HapA was nearly exclusively distributed in *Geng* rice. The difference between HapC and HapA was in 35 non-synonymous mutations (Figure 5a,d). Multiple comparisons demonstrated that HapC had a significantly lower TRN than other three haplotypes (Figure 5e). These results suggested that OsMPK15 may be an important gene regulating TRN.

### 3.6. GWAS of Rice Root Traits Reveals Abundant Epistatic Interactions

**Figure 6:**
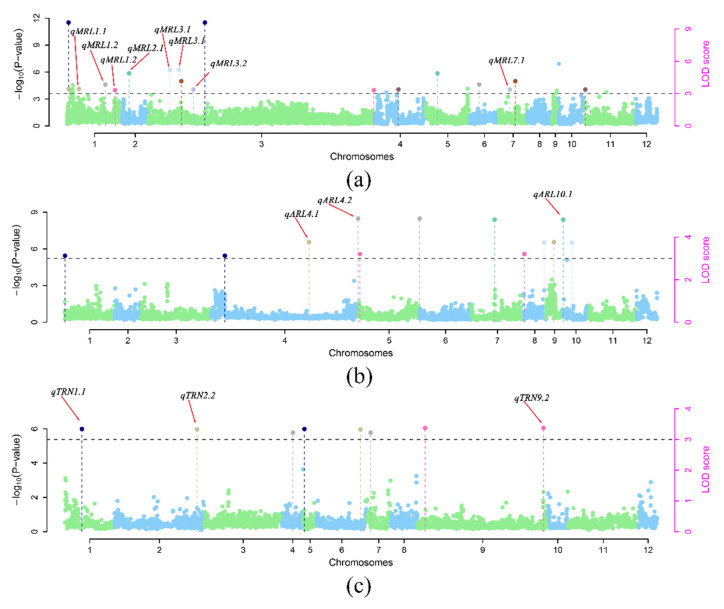
Manhattan plots of epistatic interaction among the loci of MRL, ARL, and TRN. Manhattan plots of epistatic interactions at the main effect sites of MRL (**a**), ARL (**b**), and TRN (**c**). The red arrow represents the QTLs co-localized by GLM, MLM, and epistatic interactions. The −log_10_ (*p*) value is reported on the left y-axis, which was obtained from the whole genome scanning of all markers in 3VmrMLM. The LOD score is reported on the right y-axis, which was obtained from the significant likelihood ratio test and suggested the dominant locus in the second step of 3VmrMLM, with the threshold LOD = 3.0 (black dotted line). If LOD score ≥ 20, the LOD scores obtained are transformed as LOD′ = 20 + (LOD − 20)/100. These LOD scores, along with their known genes, are shown in points with straight lines. All the main-effect genes with grey and blue characters are identified in one and at least two datasets. All the genes with underline are around suggested loci.

We used the recently developed 3VmrMLM software package to conduct epistatic interaction analysis on the GWAS (−log_10_ (*p*) > 3) results of GLM. As a result, 19 significant loci were detected in MRL (*n* = 9), ARL (*n* = 6) and TRN (*n* = 4), among which 13 were associated with GWAS results whereas six were not (Figure 6). The loci in the *qMRL3.2* region and the position of 5,013,178 bp on chromosome 1 were found to have the greatest contribution rate to MRL (10.35%) (Table 3 and Table S3). When only single loci were considered, 8, 2, and 3 QTLs were associated with MRL, ARL, and TRN, but when interactions were considered, more association loci emerged. In GWAS analysis, interaction loci are often overlooked, resulting in the absence of favorable genes. In the GWAS analysis process, when only one single locus was considered, neither of the two interacting genes had a significant effect, but the interaction between showed a significant effect. These problems could be solved by epistasis. These results show that epistatic interaction is an important part of GWAS and may help to understand the heritability of complex traits in GWAS.

## 4. Discussion

Rice root is a very important and complex system controlling rice yield and adaptation to a complex environment [61]. However, it is difficult to observe root growth since it occurs underground and to choose the ideal plants, which may be solved by indirect selection using markers closely related to genes controlling the root traits [62,63]. In this study, MRL, ARL, and TRN of 391 rice accessions were measured, and statistical epistasis and association mapping were used to excavate the excellent alleles regulating rice root system.

GWAS is a genetic survey of the entire genome to detect variations associated with traits in natural populations and is a powerful method for profiling complex traits. Its efficiency is largely determined by marker density, population size, and statistical methods [38,39]. Previously, population sizes ranging from 200 to 3000 have been used for GWAS in rice [64,65,66]. Here, we performed GWAS on 1821713 high-confidence SNPs and MRL, ARL, and TRN of 391 rice accessions using GLM and MLM from rMVP package (Supplementary Figure S1 and Table S1). Although the natural population of 391 accessions was not large enough, there were significant phenotypic variations in root traits, as the MRL ranged from 2.64 to 24.81 cm, the ARL from 1.99 to 12.38 cm, and the TRN from 2.36 to 6.86 (Table 1 and Supplementary Figure S3). These significant phenotypic variations may be associated with high genetic diversity.

Rice roots are mainly composed of seed roots, adventitious roots, and crown roots, which are of great significance to the fixation, nutrition, and water absorption of rice plants. Here, we detected 13 QTLs related to rice root traits (MRL (*n* = 8), ARL (*n* = 2), TRN (*n* = 3)) (Table 2). In reference to the previously reported QTLs and genes, three known root trait-related QTLs and genes were mapped in this study. To be specific, a gene *OsGNOM1* affecting the adventitious root formation in rice was mapped in *qMRL3.2*; a gene *OsARF12* mediating root elongation in rice was mapped in *qMRL4.1*; and a *qRL8.1* site that has been proved to affect the length of main roots in rice was mapped in *qMRL8.1*. These results confirm the reliability of GWAS in this study and can be used for further candidate gene analysis. In addition, 10 previously undescribed QTLs were identified as novel QTLs (Table 2). 

Rice root length and root growth angle are also involved in the resistance ability of rice to drought [67], which are key traits that directly affect rice yield. In this study, 12 SNPs were found in the coding region (*qMRL1.1* and *qARL1.1*) of LOC_Os01g52250 in *Xian* rice (Supplementary Figure S7 and Table S5) and the whole population (Figure 2 and Table 2), and their variations led to differences in MRL and ARL between rice accessions. In the *qMRL3.3* region, the lipoxygenase gene *OsLOX5* is the first key enzyme in the JA biosynthesis pathway [68]. JA is also a signal substance in plants and plays an important role in plant development. It has been reported that rice development is controlled by JA. Therefore, it can be speculated that *OsLOX5* also affects rice MRL through the JA pathway. We found an ethylene receptor gene *OS-ETR4* associated with MRL in the *qMRL7.1* region. The positive regulation of IAA on *OS-ETR4* mRNA level is mediated by an acetylene dependent pathway [69]. It has been reported that *OsRTH1* may be a homologue of RTE1 in rice, which regulates ethylene response in rice [70]. *OsRTH1* overexpression in rice could significantly inhibit the ethylene-induced growth and development changes and affects the development of adventitious roots. Therefore, we speculate that *OS-ETR4* is directly involved in MRL regulation by reducing ethylene sensitivity. We also identified the cloned gene *OsMYB* (*qARL9.1*) associated with rice ARL. Recent studies have reported that *OsHKT4* mutant plants are highly sensitive to salt stress. Compared with the wild type, the mutant exhibited retarded growth and reduced fresh weight and stem length. *OsMYBc* can regulate the expression of *OsHKT4* and affect the salt tolerance of rice, thereby affecting its development [71,72]. Therefore, according to our results, we speculated that *OsMYBc* regulates salt tolerance of rice by controlling ARL. These results provide a new theoretical basis for research on the regulation mechanism of rice root length in the future. It has been reported that the slender rod gene *SLR1* encodes a DELLA protein, which is a negative regulator of GA signaling [73]. It can also integrate and amplify SA- and JA-mediated defense signals, control root length and root number of rice, and play multiple roles in regulating rice growth and innate immune response. Interestingly, we found a mitogen-activated protein kinase *OsMPK15* associated with TRN in our candidate genes. LOC_Os11g17080 (*OsMPK15*) is a cloned gene on chromosome 11 and annotated as a mitogen-activated protein kinase. Knockout of *OsMPK15* can lead to the constitutive expression of disease persistence-related (PR) genes, increase the accumulation of reactive oxygen species triggered by chitin, enhance the accumulation of SA and JA, and significantly promote the resistance of rice [74]. We speculate that *OsMPK15* also regulates rice TRN by controlling SA and JA. Our results provide a reliable resource for exploring genes related to rice root length traits. In Oryza sativa, there are ancient and definitive differences between the two major subspecies, *Xian* and *Geng*, but the breeding history suggests a finer genetic architecture. In this study, there were significant differences in genetic diversity between 213 *Xian* and 147 *Geng* rice accessions. A total of 5 QTLs were detected in *Xian* and the total subpopulation, whereas no QTLs were detected in *Geng*. During the domestication process, most homozygous individuals of rice were composed of homozygous individuals, and infiltration from other populations was limited, resulting in genome diversity within and among rice populations.

Rice root is a complex system affected by genetic factors, environment, and population stratification. In GWAS, SNPs are usually detected to understand the association between the whole genome and the traits of interest. Unit point analysis is usually used to estimate the effect of single SNPs. However, it can only identify SNPs with relatively strong effects, while missing some important SNPs with minor effects [75,76]. We used the VmrMLM method to detect the epistasis of SNP loci exceeding the GLM threshold *p* = 1.0 × 10^−3^. We detected a total of 19 pairs of genes with significant epistasis, among which six were not detected in the two models of GWAS (Table 3). These genes may be new genes affecting rice roots. For a pair of genes, each individual gene may have weak or even no association with the trait, but their interaction is strongly associated with the trait. Overall, we detected more dominant loci related to rice roots by epistasis, providing abundant genetic resources for rice breeding.

In general, we obtained a set of loci significantly associated with rice root traits by GWAS and epistasis analysis of 391 rice accessions. The candidate genes were further screened by haplotype block structure analysis of SNPs that are significantly associated with agronomic traits and functional annotation of each gene. As expected, although a large linkage disequilibrium contains many SNPs in candidate regions, our results demonstrate that the number of candidate genes can be significantly reduced by combining haplotype block structure with gene function annotation. Further epistatic analysis improved the accuracy of genome prediction. Generally, our results should be helpful for future gene functional analysis and provide valuable information for rice gene cloning research.

## 5. Conclusions

Our identified genetic regions associated with root development traits and the newly identified loci may contribute to marker-assisted QTL pyramid / gene introgression breeding programs in the future to stabilize and improve rice yield. It also provides a rich source of information on the natural genetic variation in the evolution, domestication, and breeding of indica and japonica rice associated with seed root length and other adaptive traits. Future research can be focused on verifying the role of these candidate genes and their functional variations. Genetic transformation and DNA insertion mutation screening will be used to determine whether these genes affect rice root structure.

## Figures and Tables

**Table 1:** Statistics of MRL, ARL, and TRN in different rice populations.

Population	Accessions	MRL		ARL		TRN	
		Mean ± SD	Range	Mean ± SD	Range	Mean ± SD	Range
All	391	10.11 ± 13.93	2.64–24.81	6.25 ± 3.25	1.99–12.38	4.46 ± 0.53	2.33–6.86
Admix	10	12.28 ±17.28	4.94–19.20	6.88 ± 1.63	3.77–8.32	4.60 ± 0.35	3.57–5.50
Aus	21	15.14 ± 18.59	7.93–23.71	8.68 ± 3.86	4.89–11.94	3.83 ± 0.22	3.00–4.75
*Xian*	213	9.71 ± 14.75	2.64–24.81	5.88 ± 2.99	1.99–12.38	4.54 ± 0.52	2.33–6.85
*Geng*	147	9.83 ± 7.91	3.61–18.60	6.39 ± 2.62	2.63–11.03	4.44 ± 0.53	2.66–6.60

Note: MRL, main root length; ARL, ratio of total root length to TRN; TRN, total root number.

**Table 2:** Thirteen regions with significant signals related to MRL, ARL, and TRN in the genome-wide association study of 391 rice accessions.

Trait	QTL Name	Chr	Lead SNP (bp)	Model	*p* Value	Known Genes/QTLs
MRL	*qMRL1.1*	1	30,384,232	GLM	2.95 × 10^9^	
			30,384,232	MLM	9.09 × 10^8^	
	*qMRL1.2*		31,258,476	GLM	1.40 × 10^8^	
			31,258,476	MLM	1.47 × 10^6^	
	*qMRL3.1*	3	13,180,766	GLM	1.54 × 10^8^	
			13,180,766	MLM	4.81 × 10^7^	
	*qMRL3.2*		26,623,430	GLM	1.51 × 10^8^	*OsGNOM1* [59]
			26,623,430	MLM	3.70 × 10^6^	
	*qMRL3.3*		28,214,674	GLM	2.17 × 10^9^	
			28,214,674	MLM	2.84 × 10^6^	
	*qMRL4.1*	4	34,986,846	GLM	7.43 × 10^6^	*OsARF12* [60]
			34,983,993	MLM	5.16 × 10^6^	
	*qMRL7.1*	7	9,145,116	GLM	3.65 × 10^7^	
			9,145,116	MLM	3.06 × 10^6^	
	*qMRL8.1*	8	27,833,555	GLM	1.58 × 10^7^	*qRL8.1* [42]
			27,836,609	MLM	7.39 × 10^6^	
ARL	*qARL1.1*	1	30,377,471	GLM	1.13 × 10^7^	
			30,377,471	MLM	1.48 × 10^6^	
	*qARL9.1*	9	7,252,407	GLM	4.49 × 10^6^	
			7,252,407	MLM	1.77 × 10^6^	
TRN	*qTRN9.1*	9	4,342,978	GLM	2.78 × 10^6^	
			4,342,978	MLM	8.02 × 10^6^	
	*qTRN9.2*		9,220,591	GLM	5.54 × 10^6^	
			9,220,591	MLM	7.45 × 10^6^	
	*qTRN11.1*	11	9,292,368	GLM	1.73 × 10^7^	
			9,292,368	MLM	2.32 × 10^6^	

Note: MRL, main root length; ARL, ratio of total root length to TRN; TRN, total root number; Chr, chromosome; GLM, general linear model; MLM, mixed linear model.

**Table 3:** Significant epistasis loci of MRL, ARL, and TRN in 391 rice accessions.

Trait	Locus1			Locus2			LOD	Epistatic Effects				R^2^ (%)
	Chr	Position (bp)	QTL	Chr	Position (bp)	QTL		aa	ad	da	dd	
MRL	1	5,013,178		3	26,507,894	*qMRL3.1*	9.60	−1.41	0.08	−0.20	0.14	10.35
	1	7,001,841		1	30,090,719	*qMRL1.1*	3.43	−0.42	−0.33	−0.10	−1.15	2.60
	1	31,203,304	*qMRL1.2*	6	1,572,533		3.84	−0.77	0.27	0.27	0.73	5.68
	1	31734,441	*qMRL1.2*	3	35,282,140		3.32	0.56	0.35	0.39	0.36	0.65
	2	9,010,216	*qMRL2.1*	5	6,000,983		4.88	−0.75	−0.04	−0.12	0.03	2.71
	3	13,312,667	*qMRL3.1*	3	13,717,078	*qMRL3.1*	5.20	1.02	−0.02	−0.46	0.59	6.83
	3	16,205,852		7	18,854,004		4.15	−0.32	−0.38	0.51	−0.93	1.84
	3	26,394,185	*qMRL3.2*	7	9,286,846	*qMRL7.1*	3.38	−0.21	−0.18	1.06	0.19	3.24
	4	24,599,532		11	3,858,919		3.38	−0.22	−1.08	0.10	0.11	4.05
ARL	1	9,516,334		4	21,319,379		3.13	0.31	−0.03	−0.17	−0.24	3.67
	4	24,986,121	*qARL4.1*	9	11,295,970		3.76	−0.38	−0.30	0.22	−0.44	3.97
	4	34,995,706	*qARL4.2*	6	123,998		4.87	−0.39	−0.44	0.45	0.01	3.61
	5	477,888		8	3,132,953		3.20	0.42	0.23	0.25	0.38	6.21
	7	13,857,329		10	2,327,478		4.83	−0.39	0.27	0.14	0.47	9.76
	8	27,371,338		10	16,521,178	*qARL10.1*	3.74	−0.35	−0.29	−0.27	−0.16	1.13
TRN	1	18,625,457	*qTRN1.1*	5	5,532,069		3.34	−0.28	0.01	0.01	0.03	8.17
	2	24,828,145	*qTRN2.2*	6	11,122,708		3.33	−0.14	0.08	−0.05	−0.10	4.01
	4	16,531,248		7	3,551,879		3.22	0.16	−0.05	−0.13	−0.08	9.25
	9	1,505,551		9	9,584,680	*qTRN9.2*	3.37	0.13	0.04	0.18	0.18	1.56

Note: aa, additive-by-additive interaction effect; ad, additive-by-dominance interaction effect; da, dominance-by-additive interaction effect; dd, dominance-by-dominance interaction effect.

## Data Availability

Not applicable.

## Supplementary Materials

The following supporting information can be downloaded at: https://www.mdpi.com/article/10.3390/cimb44100301/s1, Figure S1: Distribution of single nucleotide polymorphisms (SNPs) and nucleotide diversity across the rice Nipponbare genome in the rice association panel; Table S1: Information of 391 rice accessions; Figure S2: LD decay distance estimated for 391 rice accessions; Table S2: MRL ARL and TRN mean comparison results; Figure S3: Histograms showing the data distribution of three traits of 391 rice accessions; Table S3: All significant loci of root traits detected by two models; Figure S4: The genome-wide association plots of MRL, ARL, and TRN in Xian rice population were plotted; Table S4: Significant SNPs above the threshold line in MLM and GLM; Figure S5: The genome-wide association plots of MRL, ARL, and TRN in Geng rice population were plotted using general linear model and mixed model methods; Table S5: The significant SNP loci in Xian and Geng rice populations; Figure S6: Venn diagram of the proportion of QTL controls for single and multiple traits. Table S6: All candidate gene annotations for non-synonymous mutations in exons of 10 QTLs; Figure S7: Haplotype analysis of LOC_Os03g22830; Figure S8: Haplotype analysis of LOC_Os03g49380; Figure S9: Haplotype analysis of LOC_Os07g15540.
