# Supplementary material for: Genome-Wide Association Study Reveals Candidate Genes for Root-Related Traits in Rice
Source: Curr Issues Mol Biol. 2022 Sep 21;44(10):4386–405. doi: 10.3390/cimb44100301 (PMC9601093; doi:10.3390/cimb44100301)
Supplement: Supplementary file 1 [file cimb-44-00301-s001.zip › Supplementary Figure.pdf]

Genome-wide association study Reveals Candidate Genes for root-related traits in Rice

Figure S1. Distribution of single nucleotide polymorphisms (SNPs) and nucleotide diversity across the rice Nipponbare genome in the rice association panel.

Figure S2. LD decay distance estimated for 391 rice accessions.

Figure S3. Histograms showing the data distribution of three traits of 391 rice accessions.

Figure S4. The genome-wide association plots of MRL, ARL and TRN in *xian* rice population were plotted using general linear model and mixed model methods.

Figure S5. The genome-wide association plots of MRL, ARL and TRN in *geng* rice population were plotted using general linear model and mixed model methods.

Figure S6. Venn diagram of the proportion of QTL controls for single and multiple traits.

Figure S7. Haplotype analysis of LOC_Os03g22830.

Figure S8. Haplotype analysis of LOC_Os03g49380.

Figure S9. Haplotype analysis of LOC_Os07g15540.

Supplementary Figure

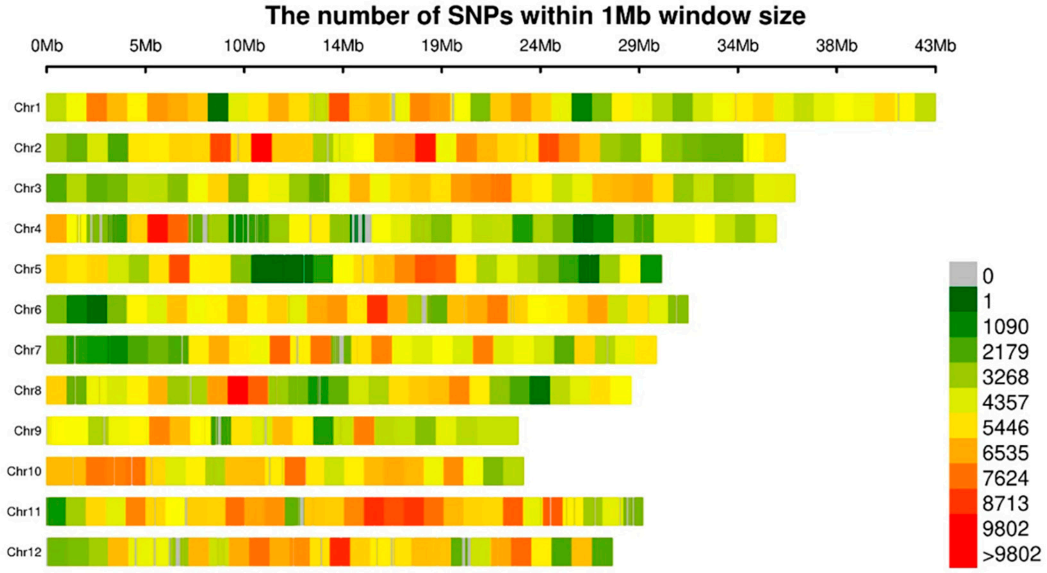

Figure S1. Distribution of single nucleotide polymorphisms (SNPs) and nucleotide diversity across the rice Nipponbare genome in the rice association panel.

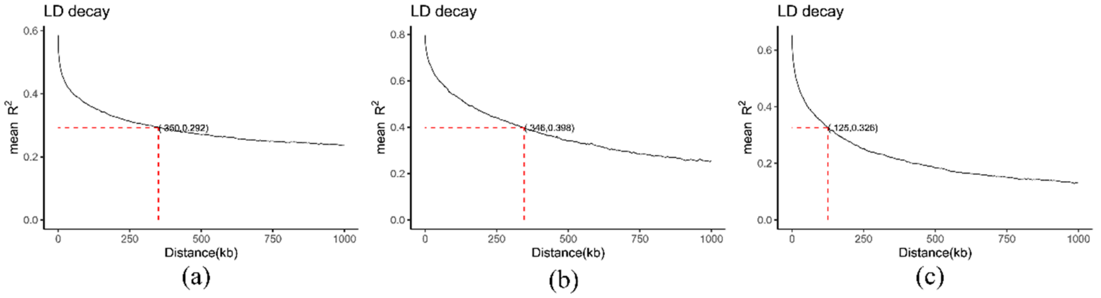

Figure S2. LD decay distance estimated for 391 rice accessions. (a) whole population; (b) geng rice accessions; (c) *xian* rice accessions.

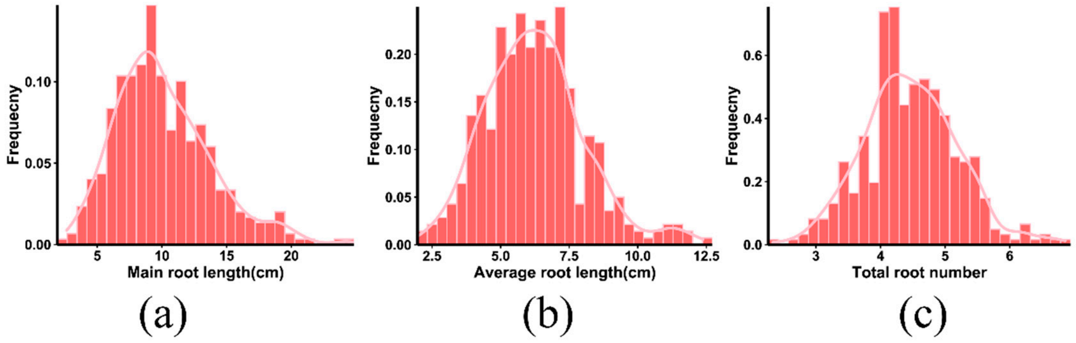

Figure S3. Histograms showing the data distribution of three traits of 391 rice accessions. (a) MRL; (b) ARL; (c) TRN.

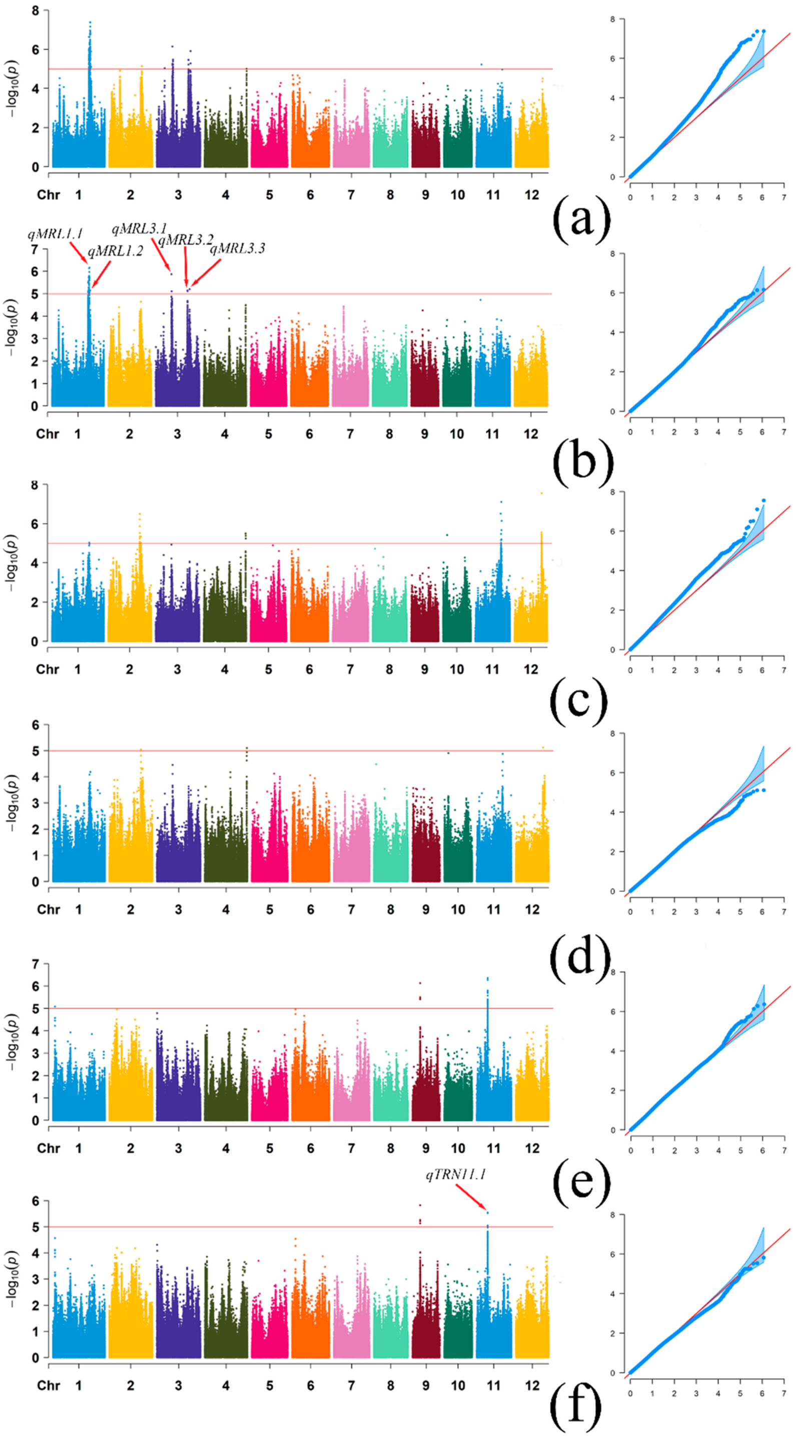

Figure S4. The genome-wide association plots of MRL, ARL and TRN in *xian* rice population were plotted using general linear model and mixed model methods. The Manhattan map of genome-wide scans showed $-\log_{10}(P)$ values corresponding to the location of each chromosome on 12 chromosomes. Red solid lines represent the whole genome significant threshold $P = 5.0 \times 10^{-5}$. The horizontal axis in the quantile-quantile (QQ) plot represents the expected value of the -log10 transform, while the vertical axis represents the observed value of the - log10 transform. Manhattan plot and QQ plot of MRL (a) in GLM (b) and MLM; Manhattan plot and QQ plot of ARL in GLM (c) and MLM (d); Manhattan plot and QQ plot of TRN in GLM (e) and MLM (f).

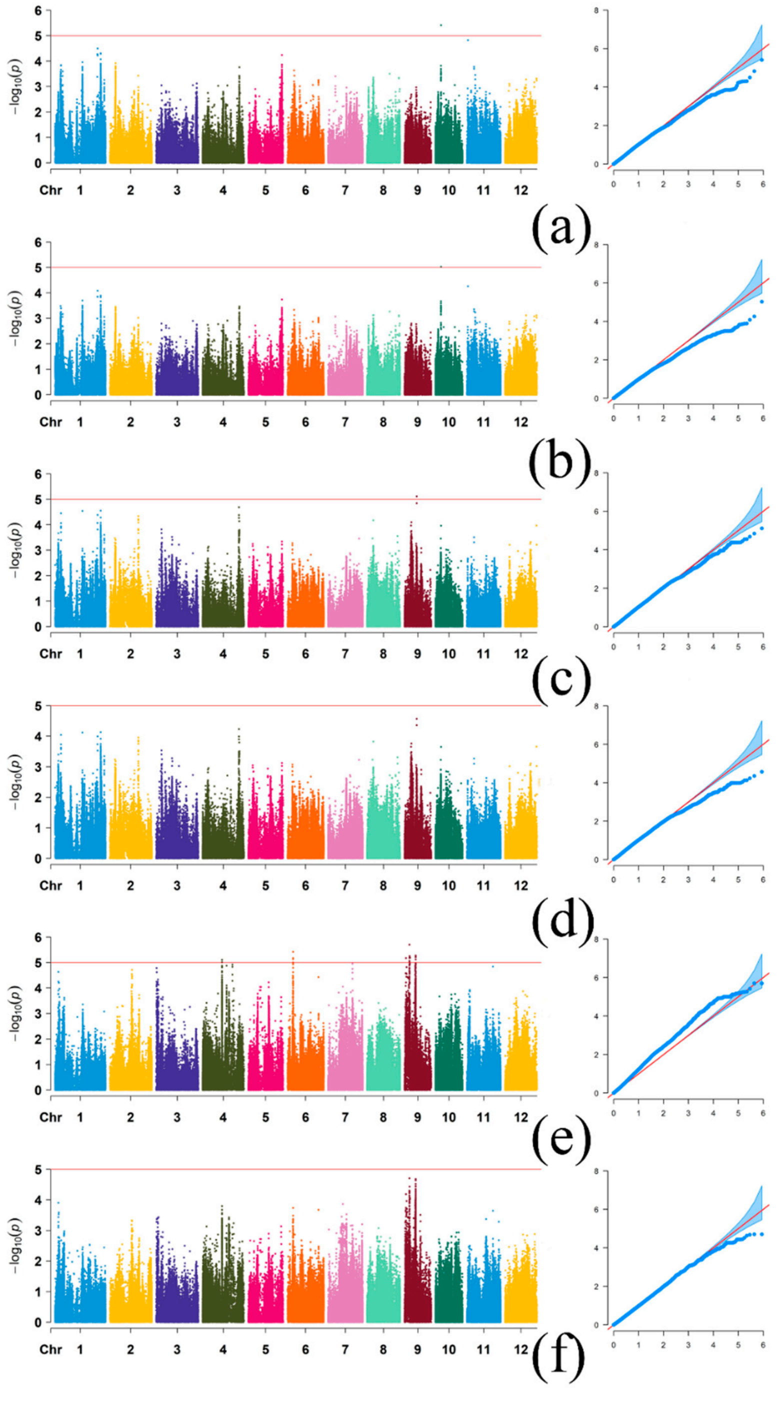

Figure S5. The genome-wide association plots of MRL, ARL and TRN in *geng* rice population were plotted using general linear model and mixed model methods. The Manhattan map of genome-wide scans showed $-log_{10}(P)$ values corresponding to the location of each chromosome on 12 chromosomes. Red solid lines represent the whole genome ' s significant threshold $P = 5.0 \times 10^{-5}$. The horizontal axis in the quantile-quantile (QQ) plot represents the expected value of the -log10 transform, while the vertical axis represents the observed value of the - log10 transform. Manhattan plot and QQ plot of MRL (a) in GLM (b) and MLM; Manhattan plot and QQ plot of ARL in GLM (c) and MLM (d); Manhattan plot and QQ plot of TRN in GLM (e) and MLM (f).

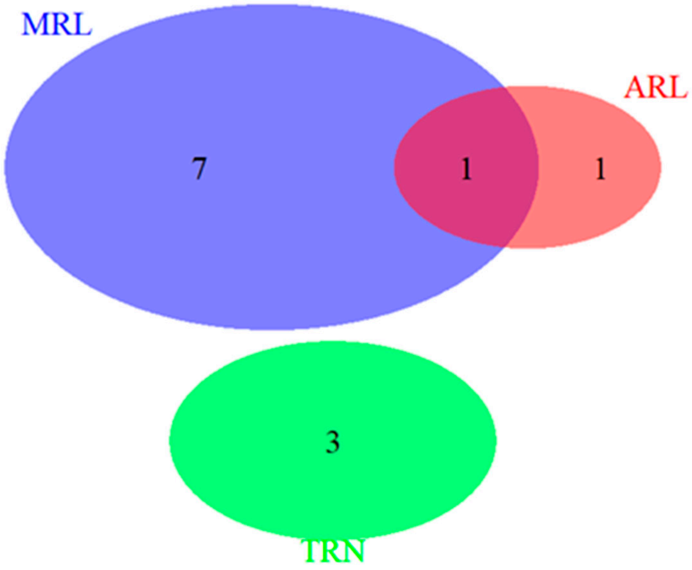

Figure S6. Venn diagram of the proportion of QTL controls for single and multiple traits.

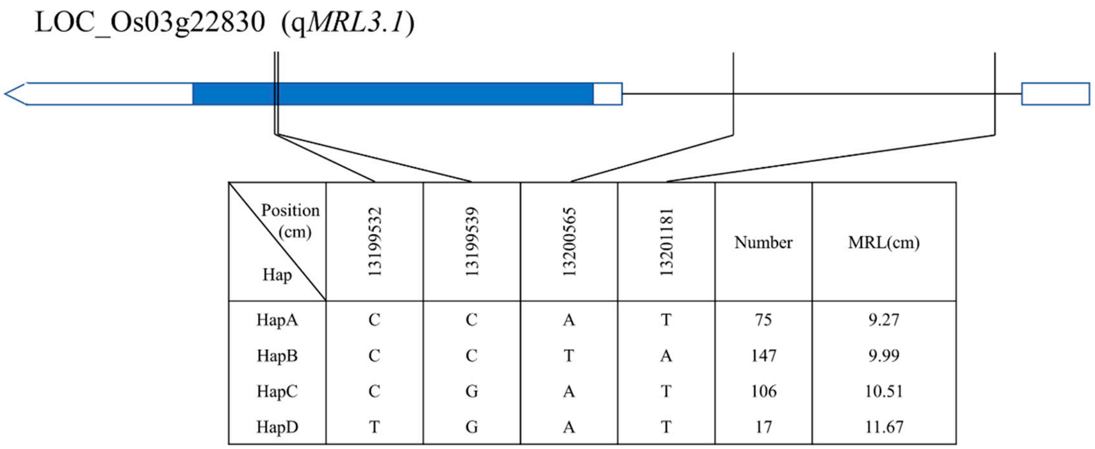

Position (cm) / Hap	13199532	13199539	13200565	13201181	Number	MRL(cm)
HapA	C	C	A	T	75	9.27
HapB	C	C	T	A	147	9.99
HapC	C	G	A	T	106	10.51
HapD	T	G	A	T	17	11.67

(a)

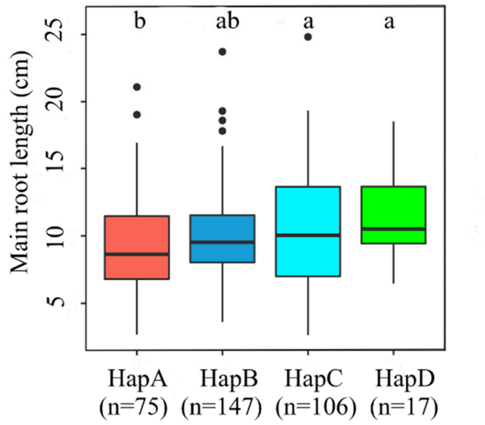

(b)

Figure S7. Haplotype analysis of LOC_Os03g22830. (a) Based on four SNPs observed in all evaluated rice

accessions, four haplotypes of LOC_Os03g22830 were identified. In the gene structure diagram of LOC_Os03g22830, the promoter is indicated by white frame; the exon is represented by blue frame; and the intron and intergenic regions are marked by black lines. A thin black line represents the genomic location of each SNP. Haplotypes with fewer than 10 accessions are not shown. (b) Based on MRL of LOC_Os03g22830 haplotype, differences between the haplotypes were statistically analyzed using Tukey's test.

LOC_Os03g49380 (q*MRL3.3*)

Position (bp) / Hap	28106907	28107105	28107212	28108390	28108491	28108646	28108964	28109489	28109540	28109736	28109769	28109888	28110040	28110837	28111227	28111886	28111909	28112240	28113053	28113079	Number	MRL(cm)
HapA	C	C	A	A	A	T	C	G	C	G	A	T	T	C	C	C	G	G	T	A	33	11.43
HapB	C	C	T	C	G	C	C	G	T	A	T	C	T	C	C	T	G	C	T	G	19	13.71
HapC	C	T	T	C	A	T	C	G	T	G	T	C	T	C	C	T	G	G	T	G	63	9.18
HapD	G	C	T	C	A	T	A	C	T	G	T	C	G	T	T	T	T	G	C	G	125	9.76
HapE	G	C	T	C	A	T	C	G	T	G	T	C	T	C	C	T	G	G	T	G	87	9.57

(a)

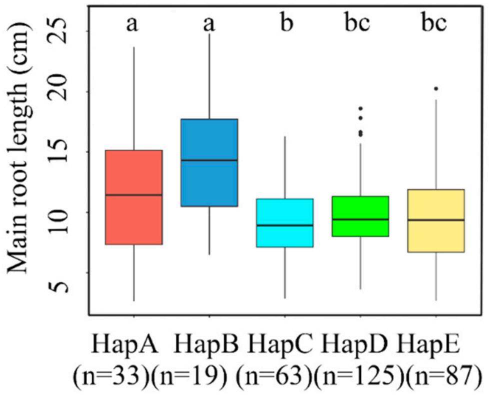

(b)

Figure S8. Haplotype analysis of LOC_Os03g49380. (a) Based on 20 SNPs observed in all evaluated rice accessions, five haplotypes of LOC_Os03g49380 were identified. In the gene structure diagram of LOC_Os03g49380, the promoter is indicated by white frame; the exon is represented by blue frame; and the intron and intergenic regions are marked by black lines. A thin black line represents the genomic location of each SNP. Haplotypes with fewer than 10 accessions are not shown. (b) Based on MRL of LOC_Os03g49380, differences between the haplotypes were statistically analyzed using Tukey's test.

LOC_Os07g15540 (q*MRL7.1*)

Position (cm) / Hap	9005325	9005432	9005554	9005679	9005705	9005741	9006340	9006496	9006526	9006562	9006588	9006901	9006908	9006930	9006976	9007031	9007152	9007205	9007276	9007388	9007680	9007805	Number	MRL(cm)
HapA	C	A	C	G	A	C	T	C	G	C	G	T	G	T	G	C	A	G	T	A	T	G	111	9.13
HapB	G	A	C	A	T	G	C	T	G	C	A	C	C	C	G	C	G	G	C	G	C	A	102	9.64
HapC	G	A	C	A	T	G	C	T	G	C	G	C	C	T	G	C	G	G	C	G	C	A	52	10.91
HapD	G	G	A	G	T	C	C	C	A	T	G	C	C	T	C	T	G	C	T	G	T	A	49	11.93

(a)

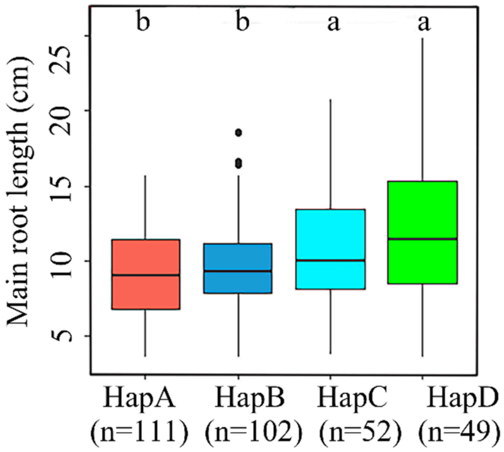

(b)

Figure S9. Haplotype analysis of LOC_Os07g15540. (a) Based on 22 SNPs observed in all evaluated rice accessions, four haplotypes of LOC_Os07g15540 were identified. In the gene structure diagram of LOC_Os07g15540, the promoter is indicated by white frame; the exon is represented by blue frame; and the intron and intergenic regions are marked by black lines. A thin black line represents the genomic location of each SNP. Haplotypes with fewer than 10 accessions are not shown. (b) Based on MRL of LOC_Os07g15540 haplotype, differences between the haplotypes were statistically analyzed using Tukey's test.
